# 

**Published:** 1887-04-23

**Authors:** 


					? ;
PRICE ONE PENNY. K-/ '
NOvSO, Vol.
''?'Ski,
APfil 23rd, 1887.
\ *?'e fSonoral PQjH>t)flice
(nof*
Per Annnm, 6s. 6d.,post free.
Monthly Parts, 6d.; post free, 7id.
Ditto per Ann., 7s. 6d., post free.

				

